# Erythrocyte parameters, anemia conditions, and sex differences are associated with the incidence of contrast-associated acute kidney injury after coronary angiography

**DOI:** 10.3389/fcvm.2023.1128294

**Published:** 2023-08-29

**Authors:** Xihong Li, Qingqing Chen, Xinrui Yang, Duanbin Li, Changqing Du, Jun Zhang, Wenbin Zhang

**Affiliations:** ^1^ Department of Clinical Laboratory, Key Laboratory of Precision Medicine in Diagnosis and Monitoring Research of Zhejiang Province, Sir Run Run Shaw Hospital, Zhejiang University School of Medicine, Hangzhou, China; ^2^ Department of Cardiology, Zhejiang Hospital, Zhejiang University School of Medicine, Hangzhou, China; ^3^ Department of Cardiology, Hangzhou Lin’an People's Hospital, Hangzhou, China; ^4^ Department of Cardiology, Key Laboratory of Cardiovascular Intervention and Regenerative Medicine of Zhejiang Province, Sir Run Run Shaw Hospital, Zhejiang University School of Medicine, Hangzhou, China

**Keywords:** contrast associated acute kidney injury, erythrocyte parameter, sex difference, anemia, coronary angiography

## Abstract

**Objective:**

Contrast-associated acute kidney injury (CA-AKI) is a critical complication when applying contrast medium, and the risk factors of CA-AKI have not been fully clarified. This study aimed to explore the relationships of CA-AKI with erythrocyte parameters, anemia conditions, and sex differences in patients after coronary angiography (CAG).

**Methods:**

In this retrospective study, 4,269 patients who underwent CAG were enrolled. CA-AKI was defined as an increase in plasma creatinine of at least 0.5 mg/dl (44 μmol/L) or 25% within 72 h after exposure to the contrast medium. Three erythrocyte parameters, including hemoglobin, hematocrit, and red blood cell (RBC) count, were collected on admission. Logistic regression analyses were used to examine the associations of sex differences and erythrocyte parameters with CA-AKI in the overall population, restricted cubic splines to visualize these associations flexibly. Moreover, stratified and sensitivity analyses were conducted to assess the robustness of the findings.

**Results:**

Overall, the mean (± standard deviations) age of patients was 67.05 ± 10.77 years, and 759 subjects (17.8%) developed CA-AKI. The results showed L-shaped relationships between erythrocyte parameters and CA-AKI incidence in each model (all *P* < 0.001). The incidence of CA-AKI was positively associated with the severity of anemia, while it showed no significant differences among the types of anemia. Moreover, female patients undergoing CAG had a higher risk of CA-AKI than male patients. Mediation analysis verified that erythrocyte parameters exerted an indirect effect on the sex differences of CA-AKI incidences.

**Conclusion:**

In conclusion, females, perioperative anemia conditions, and lower erythrocyte parameters (hemoglobin, hematocrit, and RBC count) were verified as risk factors of CA-AKI in patients undergoing CAG. Furthermore, lower erythrocyte parameters among females exerted indirect effects on the sex differences in CA-AKI incidence.

## 1. Introduction

Contrast-associated acute kidney injury (CA-AKI) is a critical complication when applying contrast medium (CM), and it remains a significant cause of hospital-acquired kidney failure (1, 2). CA-AKI is associated with increased risks of death, cardiovascular events, renal failure, and long-term hospitalizations (3, 4). The reported incidence of CA-AKI in patients with normal renal function is not high, about 1%–2% (1, 4). However, the CA-AKI incidence can rise to 5%–50% for patients with underlying kidney diseases (4, 5). When certain risk factors exist, the incidence of CA-AKI among patients undergoing coronary angiography (CAG) also increases (1, 5, 6). Therefore, identifying the risk factors for CA-AKI is indispensable to implementing reasonable preventive measures to achieve better clinical outcomes.

Herefore, aging, diabetes, female gender, congestive heart failure, atherosclerosis, and anemia have been stratified as risk factors for CA-AKI (6, 7). Most of these risk factors are irreversible, while anemia is a relatively common disease and can be reversible in most cases (8, 9). Several past studies mainly determined the influence of hemoglobin on CA-AKI (8–11). The impacts of other erythrocyte parameters, such as hematocrit and RBC count, on CA-AKI, as well as the type of anemia, were not fully illustrated so far. On the other hand, the feature of the female gender as a risk factor for CA-AKI has been controversial over the past few years, possibly related to the age and disease group of the study population (12–14). Thus, we systematically assessed the relationship between erythrocyte parameters and CA-AKI incidence from multiple parameters of hemoglobin, hematocrit, red blood cell (RBC) count, and RBC morphology. We also explored the impact of sex differences on CA-AKI incidence. Furthermore, whether erythrocyte parameters mediated indirect effects on the sex differences of CA-AKI incidence was also explored.

## 2. Methods

### 2.1. Study population

This retrospective study consecutively enrolled patients who underwent coronary angiography (CAG) or percutaneous coronary intervention (PCI) between March 2015 to December 2019 in Sir Run Run Shaw Hospital and its medical consortium hospitals. The flow chart of inclusion and exclusion of the study population is shown in Supplementary Figure S1. The main exclusion criteria are as follows: (1) diagnosis with severe anemia (hemoglobin< 60 g/L); Blood transfusion is required for bleeding from surgery or anemia; (2) pre-procedural eGFR under 15 ml/min/1.73 m^2^ or the end-stage renal disease requiring dialysis; (3) multiple administration of intravascular contrast agents; (4) pre-procedural concurrent use of nephrotoxic drugs; (5) patients with shock, pregnancy, and lactation; (6) patients diagnosed with malignant tumors. Finally, 4,269 patients were enrolled and divided into two groups according to the presence of CA-AKI. Clinical and laboratory characteristics were obtained through the query of electronic medical records. The study was conducted following the Declaration of Helsinki and approved by the Institutional Ethics Committee of Sir Run Run Hospital (20200803-34). All patients signed informed consent.

### 2.2. Data collection

Data were collected from the patient's medical records, including gender, age, body mass index (BMI), comorbidities, medications, smoking and alcohol habits, and primary characteristics of the CAG or PCI procedure. The serum creatinine (Scr), complete blood count (CBC), C-reactive protein (CRP), cardiac troponin I (cTnI), estimated glomerular filtration rate (eGFR), and other indicators on the admission of all patients were also recorded. Besides, serum creatinine levels were monitored at least three times within 72 h after the procedure.

### 2.3. Definitions and data interpretation

CAG or PCI procedures were conducted by experienced interventional cardiologists according to the current standard guidelines (15). Three different contrast agents were utilized, namely loversol, iopamidol, and iodixanol. The first two are categorized as low-osmolar contrast agents, whereas the latter belongs to the iso-osmolar category. The contrast doses used in the procedure were left to the discretion of cardiologists based on the specific situation of the operation. For all patients undergoing non-emergency PCI procedures, we administer 500 ml of 0.9% saline intravenously as a pre-hydration treatment 6–12 h prior to the surgery. CA-AKI was defined as an increase in plasma creatinine of at least 0.5 mg/dl (44.2 μmol/L) or 25% within 72 h after exposure to contrast material according to the diagnostic criteria of European Society of Urogenital Radiology's Contrast Media Safety Committee, provided that other causes have been ruled out (16, 17). According to the criteria of the World Health Organization, anemia is defined as a condition in which the hemoglobin concentration is below 130 g/L for males and below 120 g/L for non-pregnant females. Anemia is further classified into four severity levels: mild (90–120 g/L), moderate (60–90 g/L), severe (30–60 g/L), and very severe (<30 g/L) (18). The level of eGFR was determined by the CKD-EPI formula.

### 2.4. Statistical analysis

Continuous variables with a normal distribution were presented as the mean ± standard deviation (SD), whereas skewed ones were presented as the median ± interquartile range. The categorical variables were presented as numbers and frequency (%). Differences in continuous variables between groups were detected with the *t*-test or Mann–Whitney *U* test. Differences in categorical values were evaluated by the chi–square test or Fisher's exact test.

Multivariate logistic regression analyses were performed to evaluate the association of hemoglobin, hematocrit, and RBC count with CA-AKI incidence. We also used restricted cubic splines (RCS) to visualize these associations flexibly. Potential nonlinear associations are estimated by performing likelihood ratio tests. Considering the normal range and distribution characteristic of the erythrocyte parameters in all patients, we set the model of hemoglobin as five nodes at 160, 140, 120, 100, and 80 g/L. Similarly, the model of hematocrit was set as five nodes at 45, 40, 35, 30, and 25%, and red blood cell count model was set as three nodes at 5, 4, and 3 × 10^12^/L. The covariates were selected based on their recognized clinical significance and the risk factors identified in previous studies on CA-AKI (19–21), as follows: gender, age, diabetes, hypertension, smoking, drinking, left ventricular ejection fraction (LVEF), CRP, eGFR, cTnI, number of vessels undergoing PCI procedure, chronic total occlusion (CTO), intravascular ultrasonography (IVUS)/optical coherence tomography (OCT)/fractional flow reserve (FFR), contrast volume, pre-admission of statin, and diuretic.

We performed a test for linear trend by entering the median value of each piecewise of erythrocyte parameters as a continuous variable and showed it as the *P*-value for the trend (*P* for trend). Moreover, we performed a stratified analysis to explore whether the association between erythrocyte parameters and CA-AKI varied across gender. Additionally, we took the hemoglobin out separately and performed the sensitivity analysis, which divided anemia into mild and severe degrees and excluded greater than upper limit patients as reference. We also explored whether there were differences in the association of the anemia with different erythrocyte morphology and CA-AKI incidence. Finally, we speculated whether the higher likelihood of CA-AKI in females was mediated by erythrocyte parameters and conducted the mediation analysis (22).

The statistical analysis was performed using SPSS version 23.0 (SPSS, Chicago, Illinois, USA) and R version 3.5 (The R Foundation for Statistical Computing, Vienna, Austria). All statistical tests with a two-tailed *P* < 0.05 were considered statistically significant.

## 3. Results

### 3.1. Baseline characteristics

This study recruited 4,269 patients, and 759 (17.8%) patients were subsequently grouped as CA-AKI. Table 1 shows the baseline and angiographic characteristics of all patients. The patients in the CA-AKI group were older than the non-CA-AKI group (69.31 ± 10.68 *vs.* 66.56 ± 10.73 years, *P* < 0.001). Besides, the CA-AKI group had higher proportions of females, anemia, and diabetes than non-CA-AKI group (39.9% *vs.* 32.8%, *P* < 0.001; 55.1% *vs.* 39.4%, *P* < 0.001; 28.3 *vs*. 23.3, *P* = 0.004, respectively). For laboratory testing, the CA-AKI patients had higher baseline levels of CRP, eGFR, cTnI, and HbA1c than those in the non-CA-AKI patients (all *P* < 0.001). In contrast, the hemoglobin, hematocrit, and RBC count levels of the CA-AKI group were significantly lower than the non-CA-AKI group. The distribution of low-density lipoprotein (LDL) between the two groups was similar (2.08 (1.59–2.71) *vs.* 2.08 (1.54–2.72) mmol/L, *P* = 0.428). For Scr of baseline levels, there was no significant difference between the two groups (*P* = 0.06). Still, postoperative Scr levels were significantly higher in the CA-AKI group than in the non-CA-AKI group (112 (86.75–166.00) *vs.* 77.00 (65.00–93.00) μmol/L, *P* < 0.001). Besides, the characteristics of the CAG procedure between the two groups also had no significant difference (*P* = 0.08). However, the proportion of emergency PCI in the CA-AKI group was higher than that in the non-CA-AKI group (21.9% *vs.* 9.6%, *P* < 0.001).

**Table 1:** Baseline characteristics of patients enrolled.

	Overall	Without CA-AKI	CA-AKI	*P* value
	*n* = 4,269	*n* = 3,510	*n* = 759	
Demographic data				
Age, years	67.05 ± 10.77	66.56 ± 10.73	69.31 ± 10.68	<0.001
Female, *n* (%)	1,454 (34.1)	1,151 (32.8)	303 (39.9)	<0.001
Diabetes, *n* (%)	1,034 (24.2)	819 (23.3)	215 (28.3)	0.004
Hypertension, *n* (%)	2,716 (63.6)	2,222 (63.3)	494 (65.1)	0.377
Current smoker, *n* (%)	728 (17.1)	614 (17.5)	114 (15.0)	0.112
Current drinker, *n* (%)	652 (15.3)	557 (15.9)	95 (12.5)	0.023
BMI, kg/m^2^	24.42 ± 5.35	24.34 ± 5.39	24.81 ± 5.13	0.027
LVEF, %	59.60 ± 12.87	60.28 ± 12.69	56.45 ± 13.27	<0.001
Anemia, *n* (%)	1,802 (42.2)	1,384 (39.4)	418 (55.1)	<0.001
Laboratory testing				
Scr of baseline, μmol/L	76.00 [64.00–94.00]	76.00 [65.00–93.00]	73.00 [60.00–99.00]	0.06
Postoperative Scr, μmol/L	81.00 [67.00–103.00]	77.00 [65.00–93.00]	112.00 [86.75–166.00]	<0.001
Proportion of Scr elevation, %	5.10 [−3.90, 17.90]	1.95 [−5.70, 10.00]	43.60 [32.40, 68.25]	<0.001
Hemoglobin, g/L	127.69 ± 19.74	129.25 ± 18.88	120.49 ± 21.92	<0.001
Hematocrit, %	38.25 ± 5.76	38.68 ± 5.52	36.25 ± 6.40	<0.001
RBC count, × 10^12^/L	4.20 ± 0.66	4.25 ± 0.63	3.99 ± 0.73	<0.001
MCV, fl	91.33 ± 5.72	91.38 ± 5.65	91.12 ± 6.04	0.269
MCH, pg	30.49 ± 2.19	30.53 ± 2.16	30.31 ± 2.30	0.012
MCHC, g/L	333.77 ± 8.53	334.04 ± 8.50	332.51 ± 8.55	<0.001
LDL, mmol/L	2.08 [1.58, 2.71]	2.08 [1.59, 2.71]	2.08 [1.54, 2.72]	0.428
CRP, mg/L	2.30 [0.90, 8.00]	2.00 [0.80, 6.50]	4.40 [1.40, 16.55]	<0.001
eGFR, ml/min/1.73 m^2^	78.78 ± 23.27	79.42 ± 22.06	75.79 ± 28.03	<0.001
cTnI, ng/ml	0.00 [0.00, 0.20]	0.00 [0.00, 0.10]	0.08 [0.00, 1.06]	<0.001
HbA1c, %	6.00 [5.60, 6.80]	6.00 [5.60, 6.70]	6.10 [5.60, 7.20]	<0.001
Uric acid, μmol/L	374.08 ± 116.28	373.96 ± 111.63	374.59 ± 135.80	0.892
CAG/PCI data				
Procedure, *n* (%)				0.88
CAG without PCI	3,219 (75.4)	2,644 (75.3)	575 (75.8)	
CAG with single-vessel PCI	797 (18.7)	655 (18.7)	142 (18.7)	
CAG with multiple-vessel PCI	253 (5.9)	211 (6.0)	42 (5.5)	
Drug eluting balloon, *n* (%)	97 (2.3)	86 (2.5)	11 (1.4)	0.123
CTO, *n* (%)	258 (6.0)	214 (6.1)	44 (5.8)	0.818
IVUS/OCT/FFR, *n* (%)	441 (10.3)	390 (11.1)	51 (6.7)	<0.001
With non-coronary angiography, *n* (%)	174 (4.1)	149 (4.2)	25 (3.3)	0.271
Rotation = 1 (%)	41 (1.0)	32 (0.9)	9 (1.2)	0.619
IABP, *n* (%)	7 (0.2)	6 (0.2)	1 (0.1)	1.000
Emergency PCI, *n* (%)	503 (11.8)	337 (9.6)	166 (21.9)	<0.001
Contrast used, mg	80 [50, 130]	80 [50, 130]	80 [50, 140]	0.297
Contrast used per kilogram body weight, mg/kg	1.23 [0.86, 2.16]	1.22 [0.85, 2.16]	1.27 [0.87, 2.15]	0.457
Type of contrast agent, *n* (%)				0.001
Ioversol	196 (4.7)	181 (5.2)	15 (2.1)	
Iopamidol	2,656 (63.5)	2,170 (62.8)	486 (66.6)	
Iodixanol	1,333 (31.9)	1,104 (32.0)	229 (31.4)	
Isotonic contrast agent, *n* (%)	1,333 (31.9)	1,104 (32.0)	229 (31.4)	0.792
Medication, *n* (%)				
Statins	3,561 (83.4)	2,980 (84.9)	581 (76.5)	<0.001
Beta-blocker	2,151 (50.4)	1,745 (49.7)	406 (53.5)	0.065
ACEI or ARB	1,887 (44.2)	1,587 (45.2)	300 (39.5)	0.005
CCB	1,186 (27.8)	979 (27.9)	207 (27.3)	0.764
Diuretic	1,348 (31.6)	948 (27.0)	400 (52.7)	<0.001

Data are presented as mean ± SD or median (Q1-Q3) for continuous variables and *n* (%) for categorical variables.

BMI, body mass index; LVEF, left ventricular ejection fraction; Scr, serum creatinine; RBC, red blood cell; MCV, mean corpuscular volume; MCH, mean corpuscular hemoglobin; MCHC, mean corpuscular hemoglobin concentration; LDL, low-density lipoprotein; CRP, C-reactive protein; eGFR, estimated glomerular filtration rate; cTnI, cardiac troponin I; CAG, coronary angiography; PCI, percutaneous coronary intervention; CTO, chronic total occlusion; IVUS, intravascular ultrasonography; OCT, optical coherence tomography; FFR, fractional flow reserve; IABP, intra-aortic balloon pumping; ACEI, angiotensin-converting enzyme inhibitor; ARB, angiotensin receptor antagonist; CCB, calcium channel blocker.

### 3.2. Associations among the proportion of Scr elevation, erythrocyte parameters, and sex differences

**Figure 1:**
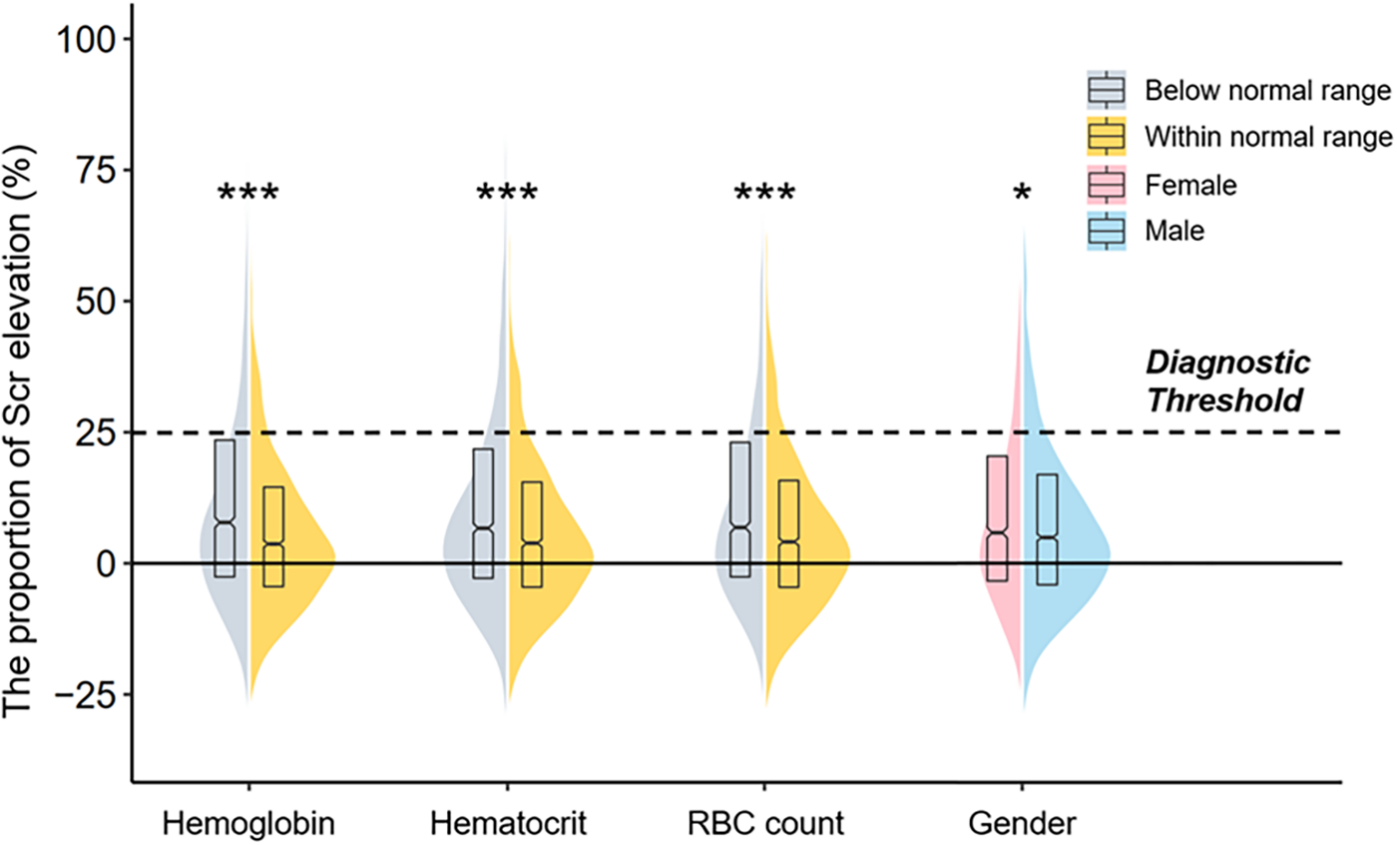
A violin plot showing the associations of the proportion of Scr elevation with sex differences and erythrocyte parameters. The dotted line means the diagnostic threshold of CA-AKI. The upper and bottom sides of the boxes indicate Q3 and Q1 separately, with cross-lines presenting the median. The bilateral curves describe the distribution of the data and the areas under the curves are equal to the amounts of different subgroups. Comparisons were performed by the Mann–Whitney *U* test, and asterisks indicate the significance levels: (***) *P* < 0.001; (*) *P* < 0.05 Scr, serum creatinine; CA-AKI, contrast associated acute kidney injury.

Postoperative Scr levels of all patients were compared to the ones on admission, and a violin plot visualized the associations of the proportion of Scr elevation with sex differences and erythrocyte parameters (Figure 1). Groups with lower hemoglobin, hematocrit, and RBC count, as well as female patients, exhibited a higher proportion of Scr elevation (all *P* < 0.05). The distribution of erythrocyte parameters across gender is shown in Supplementary Table S1. Female patients, compared with males, had lower levels of hemoglobin, hematocrit, RBC count, mean corpuscular volume (MCV), mean corpuscular hemoglobin (MCH), and mean corpuscular hemoglobin concentration (MCHC) (all *P *< 0.001). Thus, anemia was more prevalent in female patients (45.7% *vs.* 40.4%, *P* = 0.001).

### 3.3. Associations of erythrocyte parameters and sex differences with CA-AKI

**Figure 2:**
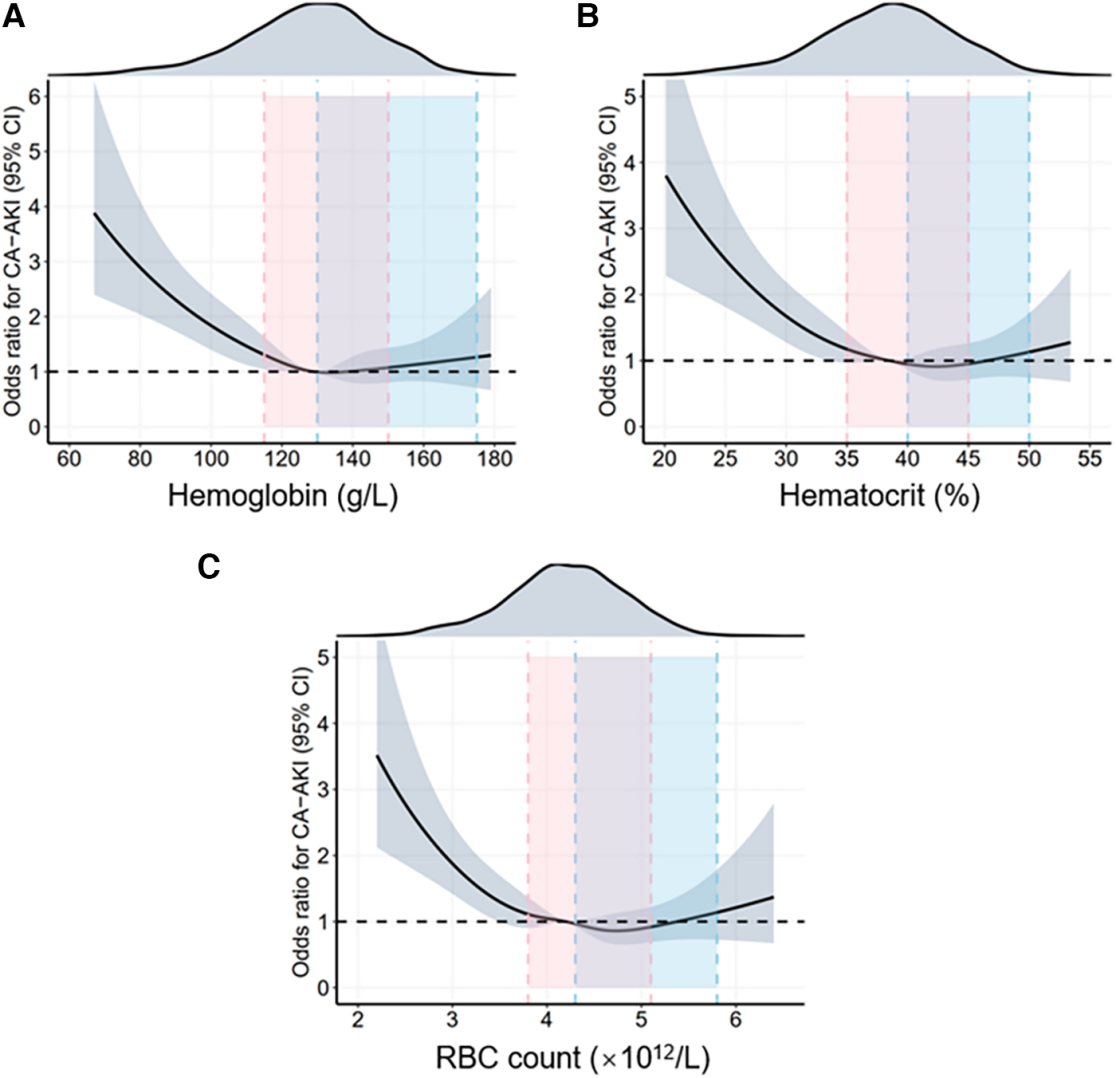
Restrictive cubic spline (RCS) analyses for exploring the association of erythrocyte parameters with CA-AKI in the overall population. The RCS model adjusted for female (yes or no), age (per 10 years), diabetes (yes or no), hypertension (yes or no), EF (< 40, 40-49, ≥50%), CRP (< 6 and ≥6 mg/L), eGFR (< 30, 30-59, 60-89, ≥90 ml/min × 1.73 m^2^), cTnI (< 0.11 and ≥0.11 ng/ml), CAG/PCI procedure (CAG without/with single-vessel/with multiple-vessel PCI), CTO (yes or no), IVUS/OCT/FFR (yes or no), volume of contrast agent (< 100 and ≥100 mg), and medications (administration of statin and diuretic) (yes or no). The solid black lines show the adjusted odds ratios of different erythrocyte parameters (**A**) for hemoglobin, (**B**) for hematocrit, (**C**) for RBC count) for CA-AKI, and the shaded areas around the solid lines indicate a 95% confidence interval of the curves. The intervals highlighted by different colors demonstrate different normal ranges for erythrocyte parameters in females and males. CA-AKI, contrast associated acute kidney injury; RBC, red blood cell.

To explore the relationship between sex differences and erythrocyte parameters with CA-AKI incidence, we conducted logistic regression analyses in the whole population, males and females, respectively. Table 2 and Supplementary Table S2 showed the results of multivariate and univariate logistic regression analysis in all patients, respectively. In this study, hemoglobin, hematocrit, and RBC count, three vital erythrocyte parameters, were separately incorporated into logistic regression analyses. Erythrocyte parameter levels were negatively correlated with CA-AKI incidence, whereas the correlations were L-shaped with *P* for trend< 0.001 in each model. Besides, the restricted cubic spline (RCS) model was built to visualize the trends above (Figure 2). For hemoglobin, hemoglobin levels< 80 mg/dl sharply increased the risk of CA-AKI (OR = 3.822, *P* < 0.001, model 1, Table 2). However, hemoglobin levels >160 mg/dl did not provide additional benefit compared to 140–160 mg/dl levels. Similar trends were consistently observed for hematocrit and RBC count before and after the covariate adjustment. In addition, all three models indicated that compared with males, females had a higher risk of CA-AKI (Model 1 (hemoglobin): adjusted-OR = 1.286, 95% CI [1.068–1.548], *P *= 0.008; Model 2 (hematocrit): adjusted-OR = 1.302, 95% CI [1.082–1.566], *P *= 0.005; Model 3 (RBC count): adjusted-OR = 1.325, 95% CI [1.107–1.586], *P *= 0.002).

**Table 2:** Multivariable logistic regression analysis of the associations of sex differences and erythrocyte parameters with CA-AKI in the overall population.

	Events/Subjects (%)	Adjusted-OR	95% CI	*P*	*P* for trend^†^
Model 1					
Hemoglobin, g/L					<0.001
[Min, 80	32/70 (45.7)	3.822	[2.158, 6.768]	<0.001	
[80, 100]	91/295 (30.8)	1.827	[1.269, 2.630]	0.001	
[100, 120]	225/975 (23.1)	1.454	[1.100, 1.921]	0.009	
[120, 140]	269/1,753 (15.3)	1.113	[0.866, 1.429]	0.404	
[140, 160]	117/1,006 (11.6)	Ref.			
[160, Max]	25/170 (14.7)	1.449	[0.894, 2.349]	0.133	
Gender					
Male	456/2,815 (16.2)	Ref.			
Female	303/1,454 (20.8)	1.286	[1.068, 1.548]	0.008	
Model 2					
Hematocrit, %					<0.001
[Min, 25]	31/88 (35.2)	2.119	[1.243, 3.614]	0.006	
[25, 30]	89/250 (35.6)	2.349	[1.643, 3.357]	<0.001	
[30, 35]	179/789 (22.7)	1.393	[1.063, 1.824]	0.016	
[35, 40]	244/1,473 (16.6)	1.153	[0.910, 1.461]	0.239	
[40, 45]	146/1,172 (12.5)	Ref.			
[45, Max]	70/497 (14.1)	1.261	[0.912, 1.743]	0.16	
Gender					
Male	456/2,815 (16.2)	Ref.			
Female	303/1,454 (20.8)	1.302	[1.082, 1.566]	0.005	
Model 3					
RBC, × 10^12^/L					<0.001
[Min, 3]	66/162 (40.7)	2.417	[1.636, 3.570]	<0.001	
[3, 4]	288/1,244 (23.2)	1.353	[1.113, 1.645]	0.002	
[4, 5]	336/2,369 (14.2)	Ref.			
[5, Max]	69/494 (14.0)	1.094	[0.811, 1.477]	0.555	
Gender					
Male	456/2,815 (16.2)	Ref.			
Female	303/1,454 (20.8)	1.325	[1.107, 1.586]	0.002	

All Models adjusted for female (yes or no), age (per 10 years), diabetes (yes or no), hypertension (yes or no), EF (< 40, 40–49, ≥50%), CRP (< 6 and ≥6 mg/L), eGFR (< 30, 30–59, 60–89, ≥90 ml/min × 1.73 m^2^), cTnI (< 0.11 and ≥0.11 ng/ml), CAG/PCI procedure (CAG without/with single-vessel/with multiple-vessel PCI), CTO (yes or no), IVUS/OCT/FFR (yes or no), volume of contrast agent (< 100 and ≥100 mg), and medications (administration of statin and diuretic) (yes or no).

Model 1, Model 2 and Model 3 were additionally adjusted for hemoglobin (< 80, 80–100, 100–120, 120–140, 140–160, ≥160 g/L), hematocrit (< 25, 25–30, 30–35, 35–40, 40–45, ≥45%), RBC count (< 3, 3–4, 4–5, ≥5*10^12^/L), respectively. RBC, red blood cell; OR, odds ratio; CI, confident interval.

^†^ This value described the trend from the minimum to the reference.

**Figure 3:**
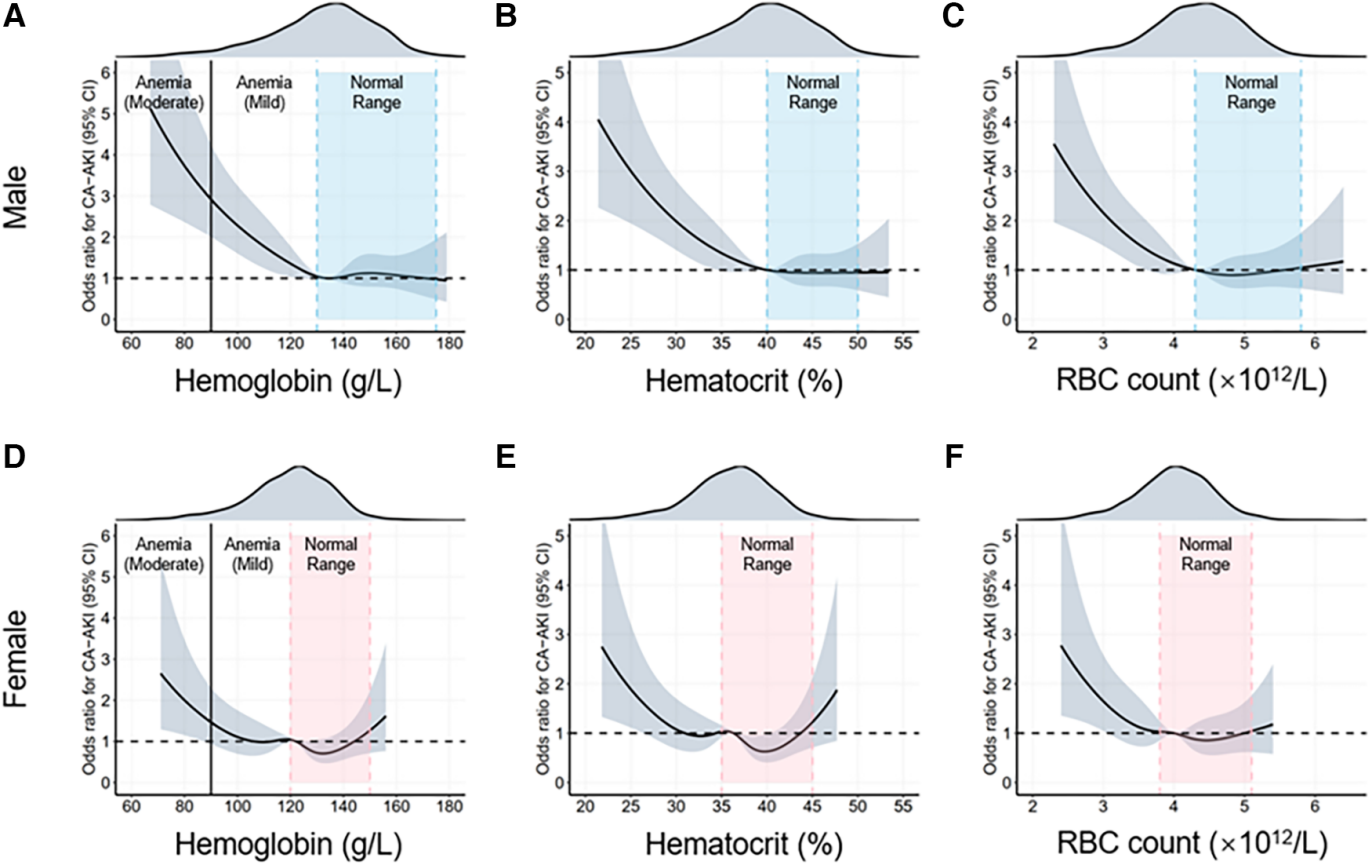
Restrictive cubic spline (RCS) analyses for exploring the association of erythrocyte parameters with CA-AKI by sex. The intervals highlighted by the dotted lines are normal ranges of different erythrocyte parameters. The adjusted odds ratios of different erythrocyte parameters for CA-AKI in the males **(A–C**) and females (**D–F**) are visualized by the black solid lines, along with 95% confidence intervals of the curve (shaded areas). CA-AKI, contrast associated kidney injury; RBC, red blood cell.

Since sex differences are closely related to CA-AKI incidence, we examined the associations between erythrocyte parameters and CA-AKI stratified by sex differences (Supplementary Tables S3,S4). Moreover, the results were similar to the multivariable logistic regression in the overall population. The RCS model was also used to visualize such trends (Figure 3).

In addition, sensitivity analysis of hemoglobin levels improved the robustness of the findings (Table 3 and Supplementary Table S5). This study demonstrated that the severity of anemia was positively associated with the incidence of CA-AKI ([moderate anemia *vs.* non-anemia]: adjusted-OR = 2.341, 95% CI [1.609–3.406], *P* for trend <0.001; [moderate anemia *vs*. normal range]: adjusted-OR = 2.364, 95% CI [1.624–3.442], *P* for trend <0.001). According to erythrocyte morphology, patients with anemia were categorized into several subgroups (Table 4). Multivariable logistic regression illustrated that increased risks of CA-AKI incidence were correlated to the microcytic anemia [OR = 1.989, 95% CI (1.053–3.757), *P* = 0.034] and normocytic anemia [OR = 1.259, 95% CI (1.012–1.566), *P *= 0.039]. However, there were no significant differences in CA-AKI incidence among various types of anemia when setting the microcytic hypochromic group as a reference (all *P* > 0.05).

**Table 3:** Multivariable logistic regression analysis of the associations of anemia and CA-AKI in the overall population.

	Events/Subjects (%)	Adjusted-OR	95% CI	*P*	*P* for trend
Model 1					
Diagnosis of anemia					
Non-anemia	341/2,467 (13.8)	Ref.			
Anemia	418/1,802 (23.2)	1.282	[1.066, 1.541]	0.008	
Model 2					
Severity of anemia					<0.001
Non-anemia	341/2,467 (13.8)	Ref.			
Anemia (mild)	348/1,621 (21.5)	1.214	[1.006, 1.465]	0.043	
Anemia (moderate)	70/181 (38.7)	2.341	[1.609, 3.406]	<0.001	
Model 3					
Severity of anemia					<0.001^†^
Greater than upper limit	9/48 (18.8)	1.521	[0.697, 3.319]	0.292	
Normal range	332/2,419 (13.7)	Ref.			
Anemia (mild)	348/1,621 (21.5)	1.225	[1.014, 1.480]	0.035	
Anemia (moderate)	70/181 (38.7)	2.364	[1.624, 3.442]	<0.001	

OR, odds ratio; CI, confident interval.

All Models adjusted for female (yes or no), age (per 10 years), diabetes (yes or no), hypertension (yes or no), EF (< 40, 40–49, ≥50%), CRP (< 6 and ≥6 mg/L), eGFR (<30, 30–59, 60–89, ≥90 ml/min × 1.73 m^2^), cTnI (<0.11 and ≥0.11 ng/ml), CAG/PCI procedure (CAG without/with single-vessel/with multiple-vessel PCI), CTO (yes or no), IVUS/OCT/FFR (yes or no), volume of contrast agent (< 100 and ≥100 mg), and medications (administration of statin and diuretic) (yes or no).

Model 1 includes the diagnosis of anemia, while the severity of anemia is incorporated into Model 2 and Model 3.

Anemia was defined as the level of hemoglobin <130 g/L in males and <120 g/L in females and categorized into mild (> 90 g/L) and moderate (60–90 g/L).

^†^ The level of hemoglobin greater than the upper limit wasn’t included.

**Table 4:** The univariable and multivariable logistic regression analysis of the associations of erythrocyte morphology and CA-AKI.

	Univariable logistic regression			*P*	Multivariable logistic regression		
	Events/Subjects (%)	OR	95% CI		OR	95% CI	*P*
Overall population							
Non-anemia	341/2,467 (13.8)	Ref.			Ref.		
Microcytic hypochromic anemia	16/80 (20.0)	1.559	[0.891, 2.728]	0.120	1.105	[0.610, 2.002]	0.741
Microcytic anemia	17/56 (30.4)	2.718	[1.520, 4.859]	0.001	1.989	[1.053, 3.757]	0.034
Normocytic anemia	212/915 (23.2)	1.880	[1.553, 2.277]	<0.001	1.259	[1.012, 1.566]	0.039
Macrocytic anemia	80/370 (21.6)	1.720	[1.309, 2.259]	<0.001	1.177	[0.870, 1.594]	0.291
Other	93/381 (24.4)	2.013	[1.552, 2.612]	<0.001	1.388	[1.040, 1.852]	0.026
Anemia population							
Microcytic hypochromic anemia	16/80 (20.0)	Ref.			Ref.		
Microcytic anemia	17/56 (30.4)	1.744	[0.791, 3.843]	0.168	1.802	[0.776, 4.183]	0.171
Normocytic anemia	212/915 (23.2)	1.206	[0.683, 2.131]	0.518	1.136	[0.620, 2.081]	0.679
Macrocytic anemia	80/370 (21.6)	1.103	[0.605, 2.013]	0.748	1.007	[0.531, 1.913]	0.982
Other	93/381 (24.4)	1.292	[0.712, 2.343]	0.400	1.219	[0.646, 2.301]	0.540

OR, odds ratio, CI, confidence interval.

Univariable logistic regression adjusted for none, and the covariables for adjustment in multivariable logistic regression analysis included female (yes or no), age (per 10 years), diabetes (yes or no), hypertension (yes or no), EF (< 40, 40–49, ≥50%), CRP (< 6 and ≥6 mg/L), eGFR (< 30, 30–59, 60–89, ≥90 ml/min × 1.73 m^2^), cTnI (< 0.11 and ≥0.11 ng/ml), CAG/PCI procedure (CAG without/with single-vessel/with multiple-vessel PCI), CTO (yes or no), IVUS/OCT/FFR (yes or no), volume of contrast agent (< 100 and ≥100 mg), and medications (administration of statin and diuretic) (yes or no).

The definition of anemia refers to Table 3.

Microcytic anemia, MCV <82 fl, MCH <27 pg, MCHC 32%–35%; normocytic anemia, MCV 82–95 fl, MCH 27–31 pg, MCHC 32%–35%; macrocytic anemia, MCV >95 fl, MCH >31 pg, MCHC 32%–35%; microcytic hypochromic anemia, MCV <82 fl, MCH <27 pg, MCHC <32%.

### 3.4. Mediation of sex differences and CA-AKI through erythrocyte parameters

**Figure 4:**
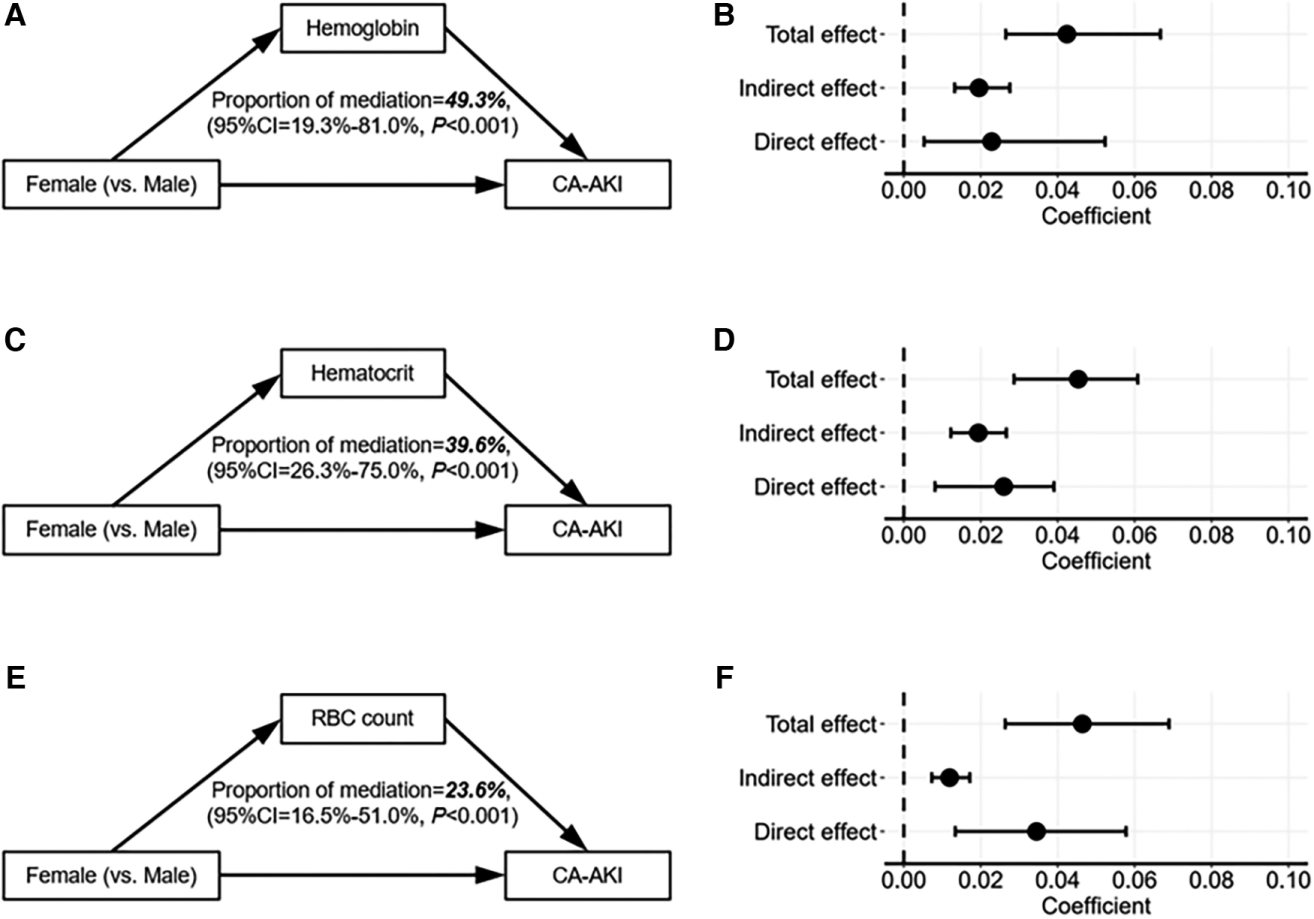
Mediation analysis of erythrocyte parameters between sex differences and the risks of CA-AKI. The mediation analyses diagram depicts the mediation effects of erythrocyte parameters on the association between sex differences and the risks of CA-AKI (**A,B**), for hemoglobin; (**C,D**), for hematocrit; (**E,F**), for RBC count). The solid arrows on the left demonstrate the relationships between variables. And on the right, the black dots indicate the coefficients of different effects, while the black lines describe the 95% confidence intervals of the coefficients. CA-AKI, contrast associated kidney injury; RBC, red blood cell; CI, confidence interval.

Subsequently, mediation analysis was conducted to explore whether erythrocyte parameters mediated sex differences in CA-AKI incidences. As shown in Figure 4, there existed a direct effect of sex difference and an indirect effect of erythrocyte parameters on the association between CA-AKI and sex differences. The result demonstrated that although the direct effect was the main contributor, three erythrocyte parameters (hemoglobin, hematocrit, and RBC count) could also exert indirect effects on the sex differences of CA-AKI incidence. To be more specific, hemoglobin showed the most significant indirect effect for the association between sex difference and CA-AKI. (hemoglobin: proportion of mediation = 49.3%, 95% CI [19.3%–81.0%], *P* < 0.001; hematocrit: proportion of mediation = 39.6%, 95% CI [26.3%–75.0%], *P* < 0.001; RBC count: proportion of mediation = 23.6%, 95% CI [16.5%–51.0%], *P* < 0.001).

## 4. Discussion

This retrospective study explored the associations of preprocedural erythrocyte parameters, anemia conditions, and sex differences with CA-AKI incidence in patients who underwent CAG/PCI in a tertiary care medical center in China. Several original observations were illustrated in this study. First, the relationship between erythrocyte parameters and CA-AKI was L-shaped, consistent across the whole population, male and female patients. Second, the risk of CA-AKI incidence was positively correlated with the severity of anemia but presented no significance among various types of anemia with different erythrocyte morphology. Moreover, we found that females had a higher risk of CA-AKI compared with males. Considering the proportion of anemia in females was significantly higher than that in men, we speculated that the higher likelihood of CA-AKI in females was mediated by lower erythrocyte parameters such as anemia. Thus, we conducted the mediation analysis to validate and quantify this mediation effect. From the mediation analysis, we found that hemoglobin, hematocrit, and RBC count mediated 49.3%, 39.6%, and 23.6% of the associations of females with CA-AKI incidence, respectively.

Several other studies observed the associations of anemia and hematocrit with CA-AKI. Sreenivasan and Murakami et al. found that anemia is associated with higher incidences of contrast-induced nephropathy (CIN) in patients undergoing coronary angiography or renal insufficiency (9, 10). Nikolsky et al. studied 6,773 patients treated with PCI and found that lower baseline hematocrit was an independent predictor of CA-AKI (23). Most of these studies only consider a single parameter of erythrocyte, while we evaluated the associations between erythrocyte parameters and the CA-AKI incidence from multiple dimensions, such as hemoglobin, hematocrit, RBC count, degree and types of anemia. To the best of our knowledge, this is the first study using RCS models to flexibly describe the relationships between erythrocyte levels (hemoglobin, hematocrit, RBC count) and CA-AKI hazard ratio. The L-shaped nonlinear relationships were observed in this study. Consistent with most previous studies, hemoglobin, hematocrit, and RBC count remained independent predictors of CI-AKI in multivariate analysis. Our findings have demonstrated that, in multivariate analysis, patients with lower hemoglobin levels exhibit a significantly elevated risk of CA-AKI compared to those with lower hematocrit and red blood cell count levels, where the disparity between the latter two is marginal. Furthermore, our mediation analysis has underscored the pronounced indirect impact of hemoglobin on the gender disparity in CA-AKI, with RBC count-mediated indirect effects being the least substantial, albeit still statistically significant. These research outcomes are consistent with the clinical application wherein hemoglobin serves as the primary indicator for anemia observation, complemented by hematocrit as a secondary measure. This alignment reinforces the argument for the effect of anemia on CA-AKI occurrence. Consequently, within the context of CA-AKI assessment, hemoglobin and hematocrit emerge as pivotal observational indices for monitoring the predisposing factor of anemia.

Besides, our study disclosed the positive association between the severity of anemia and CA-AKI. Sreenivasan *et al*. concluded that the severity of anemia was a strong predictor of CA-AKI in three subgroups of anemia (hemoglobin at 11.1 to 13.0, 9.1 to 11.0, 7.0 to 9.0 g/dl) (9). In this study, we only analyzed two degrees of anemia, mild anemia (hemoglobin greater than 90 g/L) and moderate anemia (hemoglobin at 60–90 g/L), due to the severe anemia were excluded at enrollment. Compared with the patients without anemia or mild anemia, the patients with moderate anemia had the highest risk of CA-AKI. Similarly, we were unable to observe the relationship between erythrocytosis and the risk of CA-AKI due to the limited number of cases with elevated erythrocyte parameter levels in our enrolled patients. One study found that higher hemoglobin levels in patients with polycythemia were associated with an increased risk of one-year mortality (24). However, in the gender-stratified analysis, we can observe female patients with erythrocyte parameters exceeding the upper limit of the normal level have a higher risk of CA-AKI than those with normal levels. Both hematocrit and hemoglobin are the most important determinants of blood viscosity (25). Female patients are more likely to cause elevated blood viscosity than male patients with increased erythrocyte parameter levels (26), which leads to ischemia and hypoxia in organs and vessels, particularly in the kidneys (27).

Anemia can be categorized into microcytic, normocytic, and macrocytic anemia according to the MCV and MCHC levels. The current study found that in the whole population, microcytic and normocytic anemia were correlated with higher risks of CA-AKI. In contrast, there were no significantly different risks of CA-AKI among various types of anemia in the anemia population. The pathogenesis of normocytic anemia in patients commonly results from anemia of chronic disease (ACD), such as chronic kidney disease (28, 29). A previous study has shown that anemia in patients with pre-existing chronic kidney disease was at higher risk for CIN (11). As the disease progresses, microcytic anemia can further be the clinical characteristic of ACD (28).

Injection of contrast medium has direct toxic effects on renal tubular epithelial cells, causing apoptosis and necrosis. Indirect mechanisms are associated with ischemic injury due to vasoactive substances such as endothelin, nitric oxide, and prostaglandin-mediated vasomotor changes (19). Moreover, contrast agents can also increase the oxygen binding capacity of hemoglobin, resulting in impaired oxygen delivery to peripheral tissues. So patients with anemia can further reduce oxygen delivery (30). The renal medulla is extremely sensitive to hypoxia. Coupled with enhanced metabolic demand, it makes the medulla particularly vulnerable to ischemic injury (31). Declined RBC count caused by anemia affects the antioxidant functions of RBC and then aggravates renal oxidative stress (32, 33). Therefore, anemia may play a role in the risk of CA-AKI incidence. Nevertheless, it remains unclear whether corrective anemia therapy can effectively reduce the incidence of CA-AKI.

Sexual dimorphism is a recognized feature of chronic progressive nephropathy (34). The Kidney Disease Improving Global Outcomes (KDIGO) Clinical Practice Guideline states that contrary to most CKDs, the female gender is among the shared susceptibility factors that confer a higher risk of AKI (35, 36). However, in recent years, human observational studies have reported inconsistent findings. Neugarten et al. conducted a meta-analysis of AKI studies of nearly 240 million AKI patients published between January 1978 and April 2018 (14). Their findings contradicted the existing view that the female sex confers a higher risk of AKI and instead showed a protective role. Barbieri *et al*. studied 2,851 patients undergoing CAG/PCI and found that the female gender was associated with an increased risk of CA-AKI. However, this finding was not confirmed after correcting the baseline confounders (12).

In this study, we found that females were a 1.3 times chance of developing CA-AKI than males after correction for baseline confounders, which was in line with many findings (6, 37). There are several possible explanations for the results. It has been suggested that baseline nephropathy immediately puts patients at a higher risk of developing CA-AKI. According to Chen et al., 74% of females and 45% of males have stage 3 chronic kidney disease (38). In addition, Sidhu et al. showed that the incidence of CIN was highest in older females (aged from 65 to 79 years) (39). In contrast, the association between females and CIN was insignificant in younger patients. Those are partly consistent with the results of our study. The average age of our enrolled population was 67.05 years, mainly the elderly. Previous studies have shown that in the experimental model of ischemic AKI, estrogen has a protective role in the process of kidney disease, while androgen has a harmful effect (40, 41). As sex hormone levels decline in elderly adults, this may also be one of the reasons why older females have a higher risk of CA-AKI than males. Moreover, Nikolsky *et al*. found that females tend to be more anemic than males before angiography and have a higher risk of bleeding (23). This feature was also revealed in our study. We further used mediation analysis to demonstrate the role of decreased hemoglobin, hematocrit, and RBC count in the association between women and the risk of developing CA-AKI.

Despite the important findings being mentioned, several limitations need to be recognized. First, as a retrospective study, the inherent bias of this study was unavoidable, such as perioperative blood loss leading to reduced hemoglobin levels might have influenced the occurrence of CA-AKI. Prospective multicenter research on a larger scale is necessary to support these findings. Second, the hydration status, dehydration, and fluid overload may result in a fluctuation of hemoglobin and hematocrit in patients (42). The difference in pre-procedural hydration therapy between emergency and non-emergency patients may serve as a potential bias in our study. Third, information about the etiology of anemia was not provided in our study. Given the possibility that the etiology of anemia may influence the incidence of CA-AKI to some extent, a prospective study could be considered for the etiology of anemia and exploration of its relationship with CA-AKI (8). Moreover, our study population did not include patients with severe anemia, so the association between severe anemia and the risk of developing CA-AKI cannot be fully determined.

In conclusion, females, anemia condition, and lower erythrocyte parameters (hemoglobin, hematocrit, and RBC count) significantly increased the CA-AKI risk in CAD patients undergoing CAG/PCI. Moreover, this study proved that lower erythrocyte parameters among females exerted indirect effects on the sex differences in CA-AKI incidence. Therefore, monitoring preprocedural erythrocyte parameters and anemia condition is vital for reducing CA-AKI incidence, especially in females.

## Data Availability

The raw data supporting the conclusions of this article will be made available by the authors, without undue reservation.
