# Supplementary material for: Erythrocyte parameters, anemia conditions, and sex differences are associated with the incidence of contrast-associated acute kidney injury after coronary angiography
Source: Front Cardiovasc Med. 2023 Aug 29;10:1128294. doi: 10.3389/fcvm.2023.1128294 (PMC10497172; doi:10.3389/fcvm.2023.1128294)
Supplement: Supplementary file 1 [file Datasheet1.docx]

**Supplementary Material**

**Supplementary Table 1.** The characteristics of erythrocytes in different genders

**Supplementary Table 2.** The univariable logistic regression analyses of erythrocyte parameters and sex difference on the CA-AKI

**Supplementary Table 3.** The multivariable logistic regression analyses of hematologic parameters on the CA-AKI in the males

**Supplementary Table 4.** The multivariable logistic regression analyses of hematologic parameters on the CA-AKI in the females

**Supplementary Table 5.** The univariable logistic regression analyses of anemia features and gender on the CA-AKI

**Supplementary Figure 1.** Flow chart of inclusion and exclusion of study population.

CAG, coronary angiography; PCI, percutaneous coronary intervention; eGFR, estimated glomerular filtration rate; RBC, red blood cell; CA-AKI, contrast associated acute kidney injury.

**Supplementary Table 1.** The characteristics of erythrocyte features between genders

|  | Overall  (n=4269) | Female  (n=1454) | Male  (n=2815) | *P* value |
| --- | --- | --- | --- | --- |
| Hemoglobin, g/L | 127.69±19.74 | 119.69±16.06 | 131.83±20.19 | < 0.001 |
| Hematocrit, % | 38.25±5.76 | 35.95±4.72 | 39.43±5.89 | < 0.001 |
| RBC count, ×10^12^/L | 4.20±0.66 | 4.00±0.57 | 4.31±0.68 | < 0.001 |
| MCV, fL | 91.33±5.72 | 90.33±5.71 | 91.85±5.66 | < 0.001 |
| MCH, pg | 30.49±2.19 | 30.07±2.18 | 30.71±2.16 | < 0.001 |
| MCHC, g/L | 333.77±8.53 | 332.76±8.40 | 334.28±8.55 | < 0.001 |
| Anemia, n (%) | 1802 (42.2) | 665(45.7) | 1137 (40.4) | 0.001 |

RBC, red blood cell; MCV, mean corpuscular volume; MCH, mean corpuscular hemoglobin; MCHC, mean corpuscular hemoglobin concentration.

**Supplementary Table 2.** The univariable logistic regression analyses of hematologic parameters and gender on the CA-AKI

|  |  | Events/Subjects (%) | OR | 95% CI | *P* | *P* for trend† |
| --- | --- | --- | --- | --- | --- | --- |
|  |  |  |  |  |  |  |
| Hemoglobin, g/L | |  |  |  |  | < 0.001 |
|  | [Min, 80) | 32/70 (45.7) | 6.399 | [3.849, 10.637] | < 0.001 |  |
|  | [80, 100) | 91/295 (30.8) | 3.389 | [2.478, 4.637] | < 0.001 |  |
|  | [100, 120) | 225/975 (23.1) | 2.279 | [1.787, 2.908] | < 0.001 |  |
|  | [120, 140) | 269/1753 (15.3) | 1.377 | [1.092, 1.738] | 0.007 |  |
|  | [140, 160) | 117/1006 (11.6) | Ref. |  |  |  |
|  | [160, Max] | 25/170 (14.7) | 1.31 | [0.822, 2.088] | 0.256 |  |
| Hematocrit, % | |  |  |  |  | < 0.001 |
|  | [Min, 25) | 31/88 (35.2) | 3.822 | [2.388, 6.118] | < 0.001 |  |
|  | [25, 30) | 89/250 (35.6) | 3.885 | [2.845, 5.305] | < 0.001 |  |
|  | [30, 35) | 179/789 (22.7) | 2.062 | [1.621, 2.623] | < 0.001 |  |
|  | [35, 40) | 244/1473 (16.6) | 1.395 | [1.118, 1.741] | 0.003 |  |
|  | [40, 45) | 146/1172 (12.5) | Ref. |  |  |  |
|  | [45, Max] | 70/497 (14.1) | 1.152 | [0.848, 1.565] | 0.365 |  |
| RBC, ×10^12^/L | |  |  |  |  | < 0.001 |
|  | [Min, 3) | 66/162 (40.7) | 4.16 | [2.979, 5.809] | < 0.001 |  |
|  | [3, 4) | 288/1244 (23.2) | 1.823 | [1.530, 2.172] | < 0.001 |  |
|  | [4, 5) | 336/2369 (14.2) | Ref. |  |  |  |
|  | [5, Max] | 69/494 (14.0) | 0.982 | [0.743, 1.299] | 0.900 |  |
| Gender | |  |  |  |  |  |
|  | Male | 456/2815 (16.2) | Ref. |  |  |  |
|  | Female | 303/1454 (20.8) | 1.362 | [1.159, 1.600] | < 0.001 |  |

†This value described the trend from minimum to the reference.

RBC, red blood cell; OR refers to odds ratio, CI indicates confidence interval.

**Supplementary Table 3.** The multivariable logistic regression analyses of hematologic parameters on the CA-AKI in the males

|  |  |  | Events/Subjects (%) | OR | 95% CI | *P* | *P* for trend† |
| --- | --- | --- | --- | --- | --- | --- | --- |
| Model 1 | |  |  |  |  |  |  |
|  | Hemoglobin, g/L | |  |  |  |  | < 0.001 |
|  |  | [Min, 80) | 19/46 (41.3) | 4.021 | [2.028, 7.972] | <0.001 |  |
|  |  | [80, 100) | 51/166 (30.7) | 2.276 | [1.480, 3.501] | <0.001 |  |
|  |  | [100, 120) | 114/463 (24.6) | 1.710 | [1.272, 2.299] | <0.001 |  |
|  |  | [120, 130) | 70/462 (15.2) | 1.033 | [0.753, 1.419] | 0.840 |  |
|  |  | [130, 157]‡ | 202/1659 (12.2) | Ref. |  |  |  |
|  |  | (175, Max] | 0/19 (0) | N/A | N/A | N/A |  |
| Model 2 | |  |  |  |  |  |  |
|  | Hematocrit, % | |  |  |  |  | < 0.001 |
|  |  | [Min, 25) | 18/56 (32.1) | 2.306 | [1.175, 4.523] | 0.015 |  |
|  |  | [25, 30) | 50/140 (35.7) | 2.861 | [1.825, 4.488] | <0.001 |  |
|  |  | [30, 35) | 88/374 (23.5) | 1.591 | [1.143, 2.213] | 0.006 |  |
|  |  | [35, 40) | 131/837 (15.7) | 1.111 | [0.849, 1.454] | 0.444 |  |
|  |  | [40, 50]‡ | 162/1349 (12.0) | Ref. |  |  |  |
|  |  | (45, Max] | 7/59 (11.9) | 0.876 | [0.382, 2.010] | 0.754 |  |
| Model 3 | |  |  |  |  |  |  |
|  | RBC, ×10^12^/L | |  |  |  |  | < 0.001 |
|  |  | [Min, 3) | 35/98 (35.7) | 2.085 | [1.232, 3.527] | 0.006 |  |
|  |  | [3, 4.3) | 226/1148 (19.7) | 1.302 | [1.026, 1.652] | 0.030 |  |
|  |  | [4.3, 5.8]‡ | 191/1531 (12.5) | Ref. |  |  |  |
|  |  | (5.8, Max] | 4/38 (10.5) | 0.826 | [0.277, 2.458] | 0.731 |  |

‡ normal range

†This value described the trend from minimum to the reference.

The covariables for adjustment in multivariable logistic regression analysis refer to Table 2.

RBC, red blood cell; OR refers to odds ratio, CI indicates confidence interval.

**Supplementary Table 4.** The multivariable logistic regression analyses of hematologic parameters on the CA-AKI in the females

|  |  |  | Events/Subjects (%) | OR | 95% CI | *P* | *P* for trend† |
| --- | --- | --- | --- | --- | --- | --- | --- |
| Model 1 | |  |  |  |  |  |  |
|  | Hemoglobin, g/L | |  |  |  |  | < 0.001 |
|  |  | [Min, 80) | 13/24 (54.2) | 3.423 | [1.361, 8.605] | 0.009 |  |
|  |  | [80, 100) | 40/129 (31.0) | 1.299 | [0.812, 2.077] | 0.275 |  |
|  |  | [100, 115) | 83/329 (25.2) | 1.330 | [0.947, 1.867] | 0.100 |  |
|  |  | [115, 150]‡ | 158/943 (16.8) | Ref. |  |  |  |
|  |  | (150, Max] | 9/29 (31.0) | 2.470 | [1.025, 5.952] | 0.044 |  |
| Model 2 | |  |  |  |  |  |  |
|  | Hematocrit, % | |  |  |  |  | < 0.001 |
|  |  | [Min, 30) | 52/142 (36.6) | 1.621 | [1.033, 2.543] | 0.036 |  |
|  |  | [30, 35) | 91/415 (21.9) | 1.070 | [0.771, 1.485] | 0.686 |  |
|  |  | [35, 45]‡ | 148/866 (17.1) | Ref. |  |  |  |
|  |  | (45, Max] | 12/31 (38.7) | 2.481 | [1.092, 5.633] | 0.030 |  |
| Model 3 | |  |  |  |  |  |  |
|  | RBC, ×10^12^/L | |  |  |  |  | < 0.001 |
|  |  | [Min, 3) | 31/64 (48.4) | 2.792 | [1.52, 5.128] | 0.001 |  |
|  |  | [3, 3.8) | 93/376 (24.7) | 1.286 | [0.931, 1.776] | 0.127 |  |
|  |  | [3.8, 5.1]‡ | 167/974 (17.1) | Ref. |  |  |  |
|  |  | (5.1, Max] | 12/40 (30.0) | 1.969 | [0.908, 4.27] | 0.086 |  |

‡ normal range

†This value described the trend from minimum to the reference.

The covariables for adjustment in multivariable logistic regression analysis refer to Table 2.

RBC, red blood cell; OR refers to odds ratio, CI indicates confidence interval.

**Supplementary Table 5.** The univariable logistic regression analyses of anemia features and gender on the CA-AKI

|  |  | Overall |  |  |  |  |
| --- | --- | --- | --- | --- | --- | --- |
|  |  | Events/Subjects (%) | OR | 95% CI | *P* | *P* for trend |
| Diagnosis of anemia | |  |  |  |  |  |
|  | Non-anemia | 341/2467 (13.8) | Ref. |  |  |  |
|  | Anemia | 418/1802 (23.2) | 1.883 | [1.607, 2.206] | < 0.001 |  |
| Severity of anemia | |  |  |  |  | < 0.001 |
|  | Non-anemia | 341/2467 (13.8) | Ref. |  |  |  |
|  | Anemia (mild) | 348/1621 (21.5) | 1.704 | [1.446, 2.010] | < 0.001 |  |
|  | Anemia (moderate) | 70/181 (38.7) | 3.932 | [2.854, 5.416] | < 0.001 |  |
| Severity of anemia | |  |  |  |  | < 0.001 |
|  | Greater than upper limit | 9/48 (18.8) | 1.451 | [0.696, 3.022] | 0.321 |  |
|  | Normal range | 332/2419 (13.7) | Ref. |  |  |  |
|  | Anemia (mild) | 348/1621 (21.5) | 1.718 | [1.456, 2.028] | < 0.001 |  |
|  | Anemia (moderate) | 70/181 (38.7) | 3.964 | [2.876, 5.463] | < 0.001 |  |

The definitions and severity of anemia refer to Table 3

Anemia was defined as the level of hemoglobin < 130 g/L in males and < 120 g/L in females and categorized into mild (> 90 g/L) and moderate (60-90 g/L).

OR refers to odds ratio, CI indicates confidence interval.

**Supplementary Figure 1.** Flow chart of inclusion and exclusion of study population


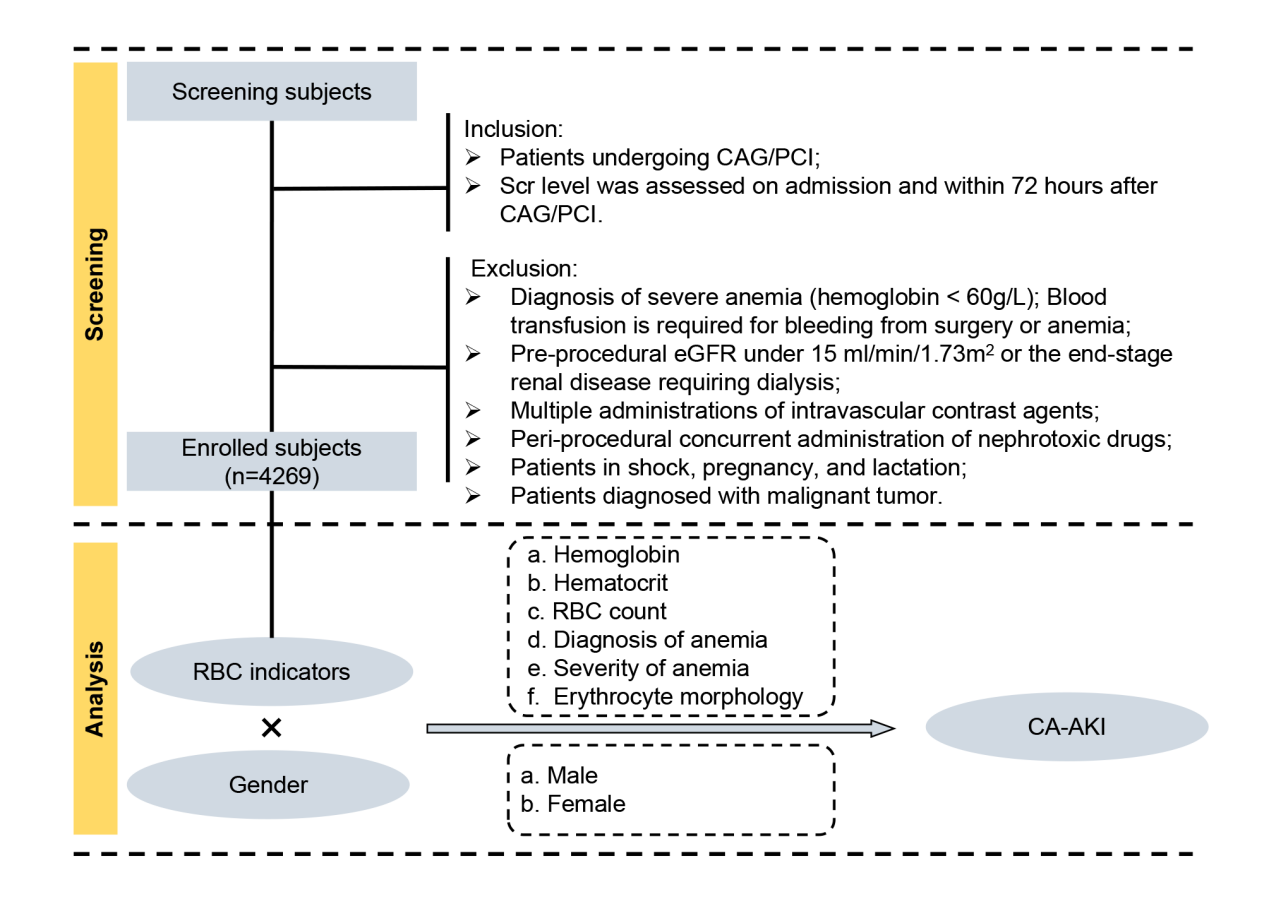


CAG, coronary angiography; PCI, percutaneous coronary intervention; eGFR, estimated glomerular filtration rate; RBC, red blood cell; CA-AKI, contrast associated acute kidney injury.
